# Assessment of CBCT–based synthetic CT generation accuracy for adaptive radiotherapy planning

**DOI:** 10.1002/acm2.13737

**Published:** 2022-10-05

**Authors:** Christopher J. O'Hara, David Bird, Bashar Al‐Qaisieh, Richard Speight

**Affiliations:** ^1^ Leeds Cancer Centre Leeds Teaching Hospitals NHS Trust Leeds UK

**Keywords:** adaptive radiotherapy, CBCT, CBCT dosimetric accuracy, CBCT‐based sCT, synthetic CT

## Abstract

**Purpose:**

Cone‐beam CT (CBCT)–based synthetic CT (sCT) dose calculation has the potential to make the adaptive radiotherapy (ART) pathway more efficient while removing subjectivity. This study assessed four sCT generation methods using 15 head‐and‐neck rescanned ART patients. Each patient's planning CT (pCT), rescan CT (rCT), and CBCT post‐rCT was acquired with the CBCT deformably registered to the rCT (dCBCT).

**Methods:**

The four methods investigated were as follows: method 1—deformably registering the pCT to the dCBCT. Method 2—assigning six mass density values to the dCBCT. Method 3—iteratively removing artifacts and correcting the dCBCT Hounsfield units (HU). Method 4—using a cycle general adversarial network machine learning model (trained with 45 paired pCT and CBCT). Treatment plans were created on the rCT and recalculated on each sCT. Planning target volume (PTV) and organ‐at‐risk (OAR) structures were contoured by clinicians on the rCT (high‐dose PTV, low‐dose PTV, spinal canal, larynx, brainstem, and parotids) to allow the assessment of dose–volume histogram statistics at clinically relevant points.

**Results:**

The HU mean absolute error (MAE) and minimum dose gamma index pass rate (2%/2 mm) were calculated, and the generation time was measured for 15 patients using the rCT as the comparator. For methods 1–4 the MAE, gamma index analysis, and generation time were as follows: 59.7 HU, 100.0%, and 143 s; 164.2 HU, 95.2%, and 232 s; 75.7 HU, 99.9%, and 153 s; and 79.4 HU, 99.8%, and 112 s, respectively. Dose differences for PTVs and OARs were all <0.3 Gy except for method 2 (<0.5 Gy).

**Conclusion:**

All methods were considered clinically viable. The machine learning method was found to be most suitable for clinical implementation due to its high dosimetric accuracy and short generation time. Further investigation is required for larger anatomical changes between the CBCT and pCT and for other anatomical sites.

## 1 INTRODUCTION

Adaptive radiotherapy (ART) is an area of great interest within the radiotherapy community as it allows a patient's radiotherapy treatment to be adapted to anatomical changes throughout their treatment.^1^, ^2^ This can significantly benefit the patient by reducing the dose to normal tissue while ensuring that the targeted volume is covered.^3^, ^4^

Adaptive pathways are known to be time‐intensive, subjective and can result in patients being treated with nonoptimal plans while awaiting a new plan. Subjective decisions are required when reviewing CBCTs for suspected significant changes in patient anatomy because direct dose calculation is not possible due to the limited electron density information in the CBCT.^5^, ^6^, ^7^ Rescan CTs (rCTs) are a dosimetrically accurate option to assess changes in the patient but result in further dose being delivered to the patient and consuming more department resources. One way of optimizing the pathway is to use each routinely acquired verification CBCT as the basis of a synthetic CT (sCT), an image set with electron density information, therefore allowing direct dose calculation and CT‐like image quality.^8^

Using CBCT‐based sCTs in the ART pathway has been investigated in the literature.^5^, ^7^, ^8^, ^9^, ^10^, ^11^, ^12^, ^13^, ^14^, ^15^, ^16^, ^17^, ^18^, ^19^, ^20^, ^21^, ^22^, ^23^, ^24^, ^25^, ^26^ Previous studies assessing sCT use in CBCT ART pathways include deforming the planning CT (pCT) to the CBCT anatomy,^11^, ^20^, ^22^, ^23^ bulk density assignment of Hounsfield units (HU) values on the CBCT,^5^, ^13^, ^20^ patient‐specific HU correction curves,^14^, ^19^ and more advanced methods such as machine learning models.^7^, ^8^, ^9^, ^12^, ^15^, ^18^, ^20^, ^21^, ^24^, ^25^, ^26^

Bulk density assignment, the mapping of mass densities onto a range of CBCT intensities, and HU correction curves were evaluated by Fotina et al.^5^ This work reported on two methods of obtaining HU values from bulk density assignment and two from CBCT to CT correction curves, with the bulk density derived median doses varying by up to 2% compared to plans calculated on the pCT. The paper concluded that the bulk density assignment is a good alternative to conversion curve‐based methods for dose calculation using CBCT images.^5^

Maspero et al.^18^ assessed the performance of a bulk density assigned CBCT‐based sCT and rigid image registration (RIR) of the pCT to rCT against a cycle generative adversarial network (cycleGAN) CBCT‐based sCT model for head‐and‐neck (H&N) patients using nonclinical software. Maspero found that the cycleGAN outperformed the other sCTs in all metrics tested (mean absolute error [MAE]: 195, 63, and 51 HU for bulk density, RIR, and cycleGAN sCTs, respectively, and 2%/2‐mm gamma index pass rates: 96% and 97.8% for RIR and cycleGAN, respectively). The dose calculation accuracy and the image quality of cycleGAN sCTs were deemed sufficient to implement within the ART pathway.^18^

This study aimed to assess four methods of sCT generation, created within a commercially available treatment planning system (TPS), to establish the most suitable for clinical implementation within the ART pathway. Method 1 used deformable registration, method 2 used bulk density assignment, method 3 iteratively removed low‐frequency artifacts and assigned accurate HU, and method 4 used a machine learning model to generate sCTs. The four methods were assessed on the H&N site as this frequently requires ART due to the long fractionation regimes (up to 7 weeks), allowing for significant anatomical changes over the treatment course as well as sufficient time for treatment changes to have an effect. The authors believe this is the first report in the literature comparing techniques in a commercially available TPS.

## 2 METHODOLOGY

### 2.1 Data acquisition

Data from 60 retrospective ART patients, treated radically for H&N pharyngeal cancer, were acquired. The patients were preliminarily broken up into 2 cohorts, 15 for testing the sCT generation methods, and the remaining 45 for training the machine learning model. For the testing cohort, the pCT (required for input in methods 1, 2, and 3), rCT (used as the ground truth), and first CBCT obtained after the rCT (acquired between 3 and 7 days after the rCT) were used. The rCT was acquired during the patient's treatment after it was determined that patient would require a replan. For the training cohort, the pCT and first captured CBCT were obtained.

All CT scans used were acquired using a Philips Brilliance Big Bore CT scanner (Philips Healthcare, Amsterdam, the Netherlands) with acquisition parameters: 120 kVp, 106 mA s, and 1.2 × 1.2 × 2‐mm resolution. The CBCT scans were acquired using an Elekta XVI scanner (Elekta, Stockholm, Sweden) with acquisition parameters: 120 kVp, 20 mA s, and 1 × 1 × 1‐mm resolution with an s20 filter and had a restricted field of view, with the shoulders and the superior region of the head typically omitted compared to the CT.

Exclusion criteria included visual differences in anatomical geometry greater than 5 mm in the nasal cavity, spinal canal, and patient external between the rCT and CBCT (and pCT and CBCT for the training cohort); sufficient anatomical coverage in the CBCT scan, ensuring that at least the eyes and base of the neck were captured on the CBCT scan; feeding tubes; significant metal artifacts; and bolus. Five patients were excluded from the training cohort due to not having the required field of view. Further patient demographic information can be found in Table 1.

**TABLE 1:** Demographic information for the 15 patients in the testing cohort as well as the number of days between the rescan CT (rCT) and cone beam CT (CBCT) and the tumor site location

Patient	Sex (male/female)	Age (year)	Gap between rCT and CBCT (days)	Tumor site
STOAT1	Male	34	5	Nasopharynx
STOAT2	Male	38	3	Oropharynx
STOAT3	Male	49	7	Nasopharynx
STOAT4	Male	53	3	Oropharynx
STOAT5	Male	73	3	Oropharynx
STOAT6	Male	65	8	Hypopharynx
STOAT7	Male	61	7	Nasopharynx
STOAT8	Female	84	4	Oropharynx
STOAT9	Male	58	3	Oropharynx
STOAT10	Male	64	5	Hypopharynx
STOAT11	Female	51	7	Oropharynx
STOAT12	Male	70	7	Oropharynx
STOAT13	Male	50	5	Oropharynx
STOAT14	Female	75	4	Oropharynx
STOAT15	Male	61	6	Oropharynx

*Note*: Patient demographic data includes sex (male and female), age (years), gap between the rescan CT and first captured CBCT post rescan CT in days, and the tumor site.

### 2.2 sCT generation

#### 2.2.1 Data preparation

RayStation Research 9B (RaySearch Laboratories AB, Stockholm, Sweden) was used to produce each of the four sCTs, with the bulk density assignment sCT generation method (method 2) also being available in the clinical TPS.

The CBCT was deformably registered within the TPS^27^ to the rCT and resampled in the frame of reference of the rCT (henceforth referred to as dCBCT) using the ANACONDA algorithm. This eliminated anatomical differences between the CBCT and rCT in the created sCTs, allowing the rCT to be used as the ground truth in comparisons. Deformable registration accuracy was qualitatively assessed by an experienced medical physicist using the vector field view and image view to verify that only clinically acceptable small anatomical deformations had occurred, ensuring that no changes in structures that should remain constant throughout the treatment were deformed, such as the brain. An external contour on the dCBCT was created using an appropriate threshold and applied to the rCT and all subsequent sCTs.

All contours, excluding the external contour, were delineated on the rCT by clinicians following local protocols for the H&N treatment site for use in assessing clinical dose–volume histogram (DVH) statistics and were copied to the sCTs created in each method.

#### 2.2.2 Method 1—deforming pCT to CBCT (sCT1)

The pCT was deformably registered to the dCBCT geometry and resampled in the frame of reference of the dCBCT. The deformable registration technique used within this project was the commercially available ANACONDA technique, a combination of HU and contour based deformable registration.^27^ The correlation coefficient similarity measure was used with no controlling contours selected for the deformable registration, creating sCT1.

#### 2.2.3 Method 2—CBCT bulk density assignment (sCT2)

**FIGURE 1:**
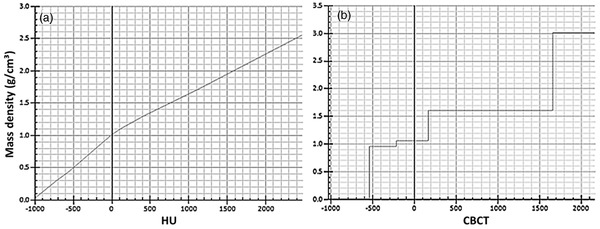
Mass density g/cm^3^ to image intensity functions: (a) the clinically used Hounsfield units (HU) to mass density g/cm^3^ function; (b) a representative example of the step function created in method 2, converting the cone‐beam CT (CBCT) intensity values to mass density g/cm^3^

The intensity distribution on the pCT was replicated on the dCBCT by manually assigning the mass density thresholds of air, lung, adipose, tissue, and cartilage/bone (0.00121, 0.26, 0.95, 1.05, 1.6, and 3 g/cm^3^, respectively) onto the image intensity range of the dCBCT. This was then compared against known structures, ensuring that bone and air had been correctly assigned based on the dCBCT image, and referring to the pCT image to ensure that other thresholds were approximately assigned correctly on the dCBCT, resulting in sCT2. Figure 1 shows the difference of the clinically used HU to mass density g/cm^3^ function compared to a representative CBCT to mass density g/cm^3^ function created using this method.

#### 2.2.4 Method 3—CBCT artifact removal and HU correction (sCT3)

An iterative correction algorithm was applied to the dCBCT to convert CBCT intensity values to HU values and remove low‐frequency artifacts.

The custom, nonclinical scripted method provided by RaySearch works iteratively to minimize the joint entropy of the two images using the same tissue peaks in the joint histogram between the dCBCT intensity values and the pCT HU values to create a conversion function. Low‐frequency artifacts were corrected by combining a mask that subtracts the dCBCT from the pCT with a mask that excludes anatomical differences between the pCT and dCBCT. The conversion function and correction map were applied to the dCBCT, creating sCT3.

#### 2.2.5 Method 4—machine learning cycleGAN model (sCT4)

The machine learning model is a custom scripted solution provided by RaySearch that is only available in the research version of RayStation and not as a module within the TPS at this current time.

Forty‐five patients were used to train a machine learning cycleGAN model and were split into training and validation cohorts consisting of 35 and 10 patients, respectively. A cycleGAN features a generator, a network that attempts to create an sCT from an input, and a discriminator, a network that attempts to determine whether an image presented to it is an sCT or a CT. These two networks work against each other, iteratively improving based on the results of the previous iteration.^28^ Each dataset within the training group had its CBCT deformably registered to the pCT and an external structure created on the CBCT. The cycleGAN model was trained over 200 epochs, minimizing the MAE of the generated sCT to the ground truth at each epoch. The trained model was used to generate sCT4.

### 2.3 Treatment plan creation

Patients were treated using a volumetric modulated arc therapy treatment (VMAT) technique. A new clinically representative 70 Gy in 35 VMAT plan was created for each of the test patients using local clinical protocols, including a 360° arc and was calculated on a 0.3‐cm^3^ dose grid. Each plan was deemed clinically acceptable by an experienced medical physicist and recalculated on each sCT within the restricted field of view on the dCBCT that excluded the shoulders and the superior region of the head. Relevant structures were copied across from the rCT to each sCT for DVH statistical analysis.

### 2.4 Analysis and testing

#### 2.4.1 Generation time

sCT generation time was measured from the start of each sCT generation method (post‐dCBCT creation) to the production of each sCT, including any necessary validation time.

#### 2.4.2 Hounsfield unit (HU) analysis

MAE was used to assess HU accuracy between each sCT and rCT within a structure clipped 15 mm from the external to prevent small surface deformable image registration (DIR) differences affecting the MAE.

#### 2.4.3 Dosimetric analysis

Dose distributions of the sCT and respective rCT were compared using global gamma index analysis with a tolerance of 2%/2 mm to allow comparison with literature. The patient external contour was clipped 15 mm from the surface to exclude dose buildup from the analysis. Only voxels with more than 20% of the prescription dose were evaluated in the gamma analysis as doses lower than this were considered clinically insignificant to the plan.

The dosimetric assessment of individual clinically relevant DVH statistics from the local clinical protocol for the H&N treatment site (high‐dose planning target volume [PTV], low‐dose PTV, spinal canal, larynx, brainstem, and parotids) was performed and compared to a clinical significance threshold of 1% that was chosen to ensure that the overall accuracy of the treatment was within the recommended ±3% absorbed dose at the dose calculation point.^29^ The structures Larynx, Parotid Lt (left), and Parotid Rt (right) were only present on some patients (11/15, 13/15, and 14/15, respectively); all other structures were present on all patients.

#### 2.4.4 Statistical methods

Generation time, MAE, and gamma index results were transformed into normally distributed data as *T*_i_ → *T*_i_^−1^, MAE_i_ → log_10_(MAE_i_), and gamma → (100‐gamma)^1⁄2^, respectively, to allow statistical analysis. The most appropriate test to determine statistical significance among the datasets was assessed using Shapiro–Wilk and Levene tests (*α* = 0.05), resulting in the use of a Welch ANOVA followed by a post hoc Games–Howell significance test for data analysis.

The DVH statistics were assessed using a linear mixed‐effects model (LMEM) to determine statistically significant systematic dose differences between the rCT and each sCT at the 95% confidence interval (CI). The LMEM was produced using STATA v15 and used: dose (normalized by prescription dose) as the dependent variable; modality, and dose statistics (*D*95%, *D*50%, *D*2%, *D*1cc, max dose, and mean dose) as fixed variables; and the patient as a random variable.

## 3 RESULTS

**FIGURE 2:**
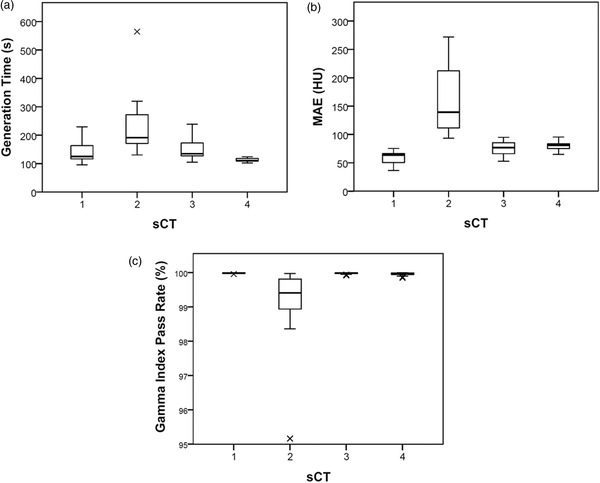
Box and whisker plots for the results of (a) the synthetic CT (sCT) generation time, (b) the mean absolute error (MAE), and (c) the gamma index pass rate for each method. Extremities of the whiskers indicate the range of the data; extremities of the box indicate the interquartile range (IQR) and line in the box indicates the median of the data. A cross indicates an outlier (third quartile + >1.5 × IQR or first quartile – >1.5 × IQR). sCT1—pCT deformable image registration (DIR) to dCBCT. sCT2—bulk density assigned dCBCT. sCT3—patient‐specific Hounsfield units (HU) calibration curve applied to dCBCT and low‐frequency artifacts removed. sCT4—trained machine learning model

Mean differences of each sCT method for the generation time, MAE, and gamma analysis are shown in Table 2 and Figure 2. It can be seen that all methods are clinically accurate, with methods 1,3, and 4 having mean MAE less than 80 HU (range: 59.7–79.4 HU), gamma pass rates more than 99.9% (range: 99.9%–100.0%), and systematic PTV dose differences less than 0.3 Gy for all analyzed structures (range: −0.10–0.29 Gy). All methods had a minimum patient gamma index of 95.2% (range: 95.2%–100.0%). Method 4 generated sCTs statistically significantly quicker (>30‐s mean time) and with a smaller standard deviation than other methods. Method 1 produced results that had statistically significantly lower MAE than all other methods (>15‐HU MAE). Method 2 was found to perform statistically significantly worse than the other methods across all measures, with statistically significant results shown in bold in Table 2.

**TABLE 2:** Mean and standard deviations of metrics tested for individual and pairwise comparisons of each synthetic CT (sCT)

	Mean (SD)				Mean differences among sCTs		
sCT method	Generation time (s)	MAE (HU)	Gamma index (%)	sCT method pairwise comparison	Generation time (s)	MAE (HU)	Gamma index (%)
sCT1	143.4 (41.2)	59.7 (10.7)	100.0 (<0.1)	sCT2–sCT1	**88.5**	**104.5**	**−0.9**
sCT2	231.9 (105.3)	164.2 (59.2)	99.1 (1.2)	sCT2–sCT3	**79.1**	**88.5**	**−0.9**
sCT3	152.9 (41.2)	75.7 (13.4)	100.0 (<0.1)	sCT2–sCT4	**119.6**	**84.8**	**−0.8**
sCT4	112.3 (6.5)	79.4 (7.7)	100.0 (0.1)	sCT3–sCT1	9.5	**16.0**	<−0.1
				sCT3–sCT4	**40.6**	−3.7	<0.1
				sCT4–sCT1	**−31.1**	**19.7**	<−0.1

*Note*: Mean and SD for the generation time, MAE, and gamma index (2%/2 mm) as well as the mean differences among sCTs for the generation time, MAE, and gamma index (2%/2 mm). Bold values indicate statistical differences determined using a post hoc Games–Howell test (*α* = 0.05). sCT1—pCT DIR to dCBCT. sCT2—bulk density assigned dCBCT. sCT3—patient‐specific HU calibration curve applied to dCBCT and low‐frequency artifacts removed. sCT4—trained machine learning model.

Abbreviations: DIR, deformable image registration; pCT, planning CT; MAE, mean absolute error; SD, standard deviation.

**FIGURE 3:**
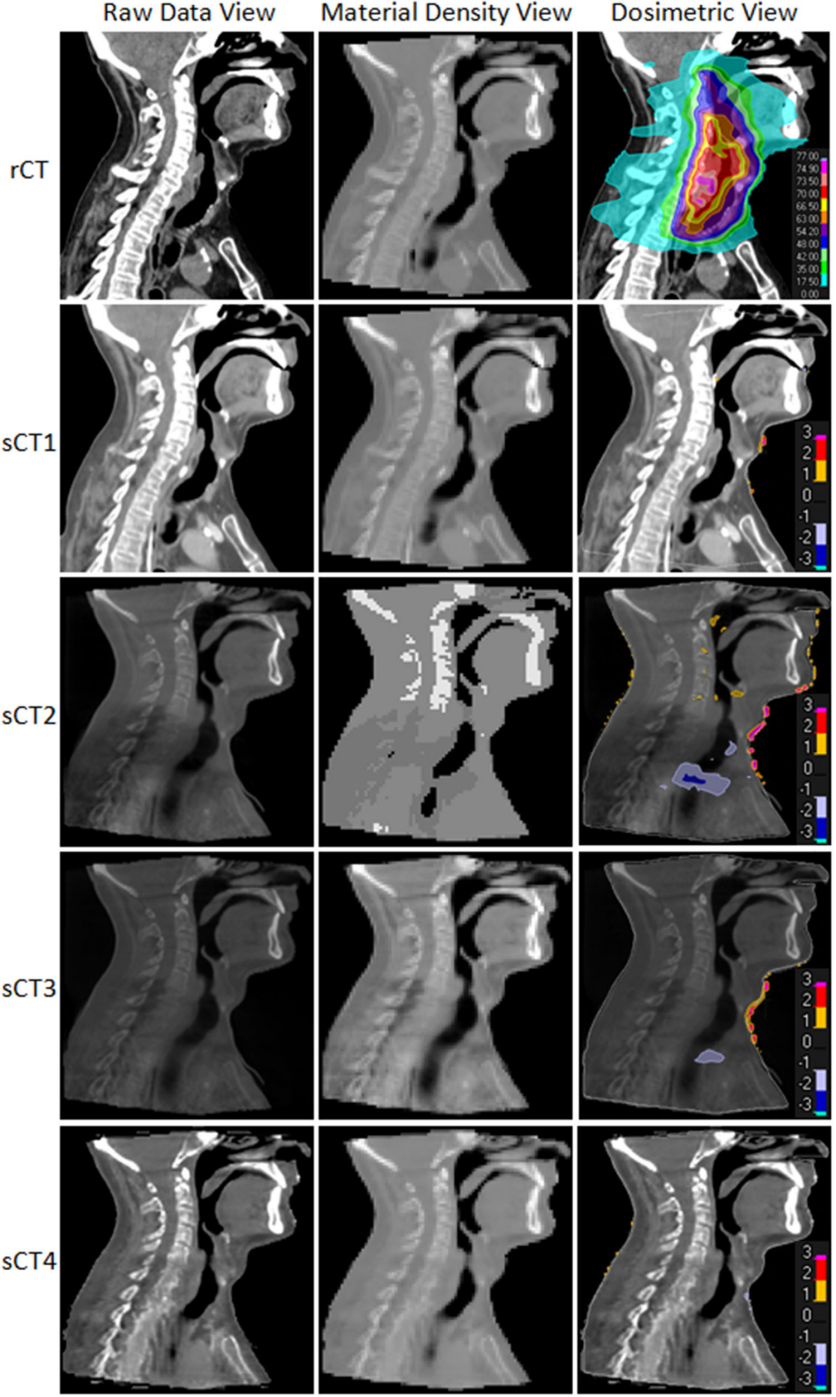
Three views of the rescan CT (rCT) and each synthetic CT (sCT) generated for a representative patient. The raw data view is typically used for contouring and general viewing of the image data. The material density view shows the mass densities on the dose grid. The dosimetric view shows the plan dose distribution for the rCT and the percentage dose difference (range −3%–3%) for the sCTs generated (normalized by the rCT dose). Positive values indicate a region is underdosed relative to the rCT and vice versa. sCT1—pCT deformable image registration (DIR) to dCBCT. sCT2—bulk density assigned dCBCT. sCT3—patient‐specific Hounsfield units (HU) calibration curve applied to dCBCT and low‐frequency artifacts removed. sCT4—trained machine learning model

Figure 3 shows representative images and dose distributions for all four sCT generation methods and highlights the dosimetric discrepancies at the boundaries found in methods 2 and 3 between high‐ and low‐mass density regions, such as in the trachea.

**FIGURE 4:**
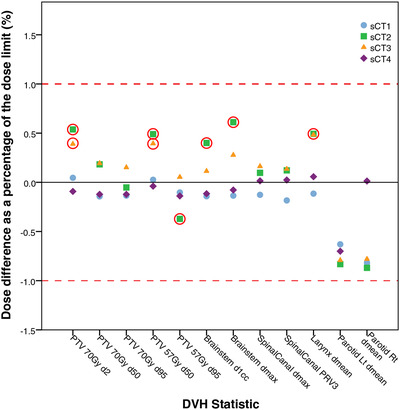
The mean percentage dose difference of the dose constraint (Table 3) for each dose–volume histogram (DVH) statistic. Results circled in red are statistically significantly different from the rescan CT (rCT) dose at the 95% confidence interval, corresponding to the results in bold in Table 3 where confidence interval ranges can also be found. Results above/below the red dotted line are clinically significant. sCT1—pCT deformable image registration (DIR) to dCBCT. sCT2—bulk density assigned dCBCT. sCT3—patient‐specific Hounsfield units (HU) calibration curve applied to dCBCT and low‐frequency artifacts removed. sCT4—trained machine learning model

Figure 4 and Table 3 show the percentage dose differences (sCT vs. rCT) of the dose constraint for each DVH statistic. Table 3 shows PTV and organ‐at‐risk percentage dose differences were between −0.37% and 0.54%, and −0.87% and 0.61% respectively at the 95% CI with statistically significant dose differences found for methods 2 and 3.

**TABLE 3:** Dose–volume histogram (DVH) statistics results summary for each synthetic CT (sCT)

	Percentage dose difference (confidence interval range)			
DVH statistic (dose constraint)	sCT1	sCT2	sCT3	sCT4
PTV 70 Gy, *D*2% (74.9 Gy)	0.05 (−0.24 to 0.33)	**0.54 (0.25–0.82)**	**0.39 (0.10–0.67)**	−0.09 (−0.38 to 0.19)
PTV 70 Gy, *D*50% (70.0 Gy)	−0.14 (−0.35 to 0.06)	0.18 (−0.02 to 0.39)	0.19 (−0.01 to 0.40)	−0.12 (−0.33 to 0.08)
PTV 70 Gy, *D*95% (66.5 Gy)	−0.13 (−0.33 to 0.06)	−0.05 (−0.25 to 0.14)	0.15 (−0.05 to 0.35)	−0.12 (−0.32 to 0.07)
PTV 57 Gy *D*50% (57.0 Gy)	0.03 (−0.24 to 0.29)	**0.49 (0.23–0.75)**	**0.39 (0.13–0.66)**	−0.04 (−0.30 to 0.22)
PTV 57 Gy, *D*95% (54.2 Gy)	−0.10 (−0.30 to 0.10)	**−0.37 (−0.57 to −0.17)**	0.05 (−0.14 to 0.25)	−0.14 (−0.34 to 0.06)
Brainstem *D*1cc (60 Gy)	−0.14 (−0.37 to 0.09)	**0.40 (0.17–0.63)**	0.11 (−0.11 to 0.34)	−0.12 (−0.34 to 0.11)
Brainstem *D*max (54 Gy)	−0.14 (−0.46 to 0.18)	**0.61 (0.29–0.93)**	0.28 (−0.04 to 0.60)	−0.08 (−0.40 to 0.24)
Spinal canal *D*max (48.0 Gy)	−0.13 (−0.40 to 0.15)	0.10 (−0.18 to 0.37)	0.16 (−0.11 to 0.44)	0.01 (−0.26 to 0.29)
Spinal canal PRV3 *D*1cc (48.0 Gy)	−0.18 (−0.41 to 0.04)	0.12 (−0.10 to 0.35)	0.14 (−0.09 to 0.36)	0.02 (−0.20 to 0.25)
Larynx *D*mean (45.0 Gy)	−0.12 (−0.43 to 0.20)	**0.49 (0.18–0.81)**	**0.48 (0.17–0.80)**	0.06 (−0.26 to 0.37)
Parotid Lt *D*mean (26.0 Gy)	−0.63 (−1.55 to 0.29)	−0.83 (−1.76 to 0.09)	−0.79 (−1.72 to 0.13)	−0.70 (−1.62 to 0.22)
Parotid Rt *D*mean (26.0 Gy)	−0.82 (−1.79 to 0.15)	−0.87 (−1.84 to 0.10)	−0.78 (−1.75 to 0.19)	0.01 (−0.96 to 0.98)

*Note*: Percentage difference in dose constraint for each method for all dose constraints ([systematic dose difference from rCT/dose constraint] × 100%) calculated using a LMEM. Bold values indicate a statistically significant result (at the 95% confidence level), with confidence interval ranges within the brackets next to each value, which have also been normalized to the dose tolerance as described earlier. No results were found to have a clinically significant difference. sCT1—pCT DIR to dCBCT. sCT2—bulk density assigned dCBCT. sCT3—patient‐specific HU calibration curve applied to dCBCT and low‐frequency artifacts removed. sCT4—trained machine learning model.

Abbreviations: DIR, deformable image registration; LMEM, linear mixed‐effects model; pCT, planning CT; PTV, planning target volume; rCT, rescan CT.

## 4 DISCUSSION

This study found that all four methods were clinically acceptable alternatives to an rCT due to the high dosimetric accuracy reported. However, method 4 was the most suitable method for clinical implementation due to its high image quality and short generation time.

Method 1 had the closest HU number to the rCT with the lowest reported MAE. Conversely, method 2 had the lowest image quality with the highest and most varied MAE and incorrectly assigned the spine as tissue (Figure 3, middle column). The incorrect assignment of the spine as tissue was a result of the compromise made when assigning the bone and tissue their respective densities due to the inaccuracies of the CBCT intensity values. The decision on the mass density threshold boundary position is highly susceptible to inter‐ and intra‐observer error, although it could potentially be improved with more clinical experience and well defined protocols. The corrections applied in method 3 improved the HU accuracy over method 2, allowing more accurate identification of bone (Figure 3, middle column). Maspero et al.^18^ and Eckl et al.^15^ reported similar MAE of 18.4 and 2.2 HU lower, respectively, compared to method 4 using a cycleGAN CBCT‐based sCT generation method. This shows that the clinically available cycleGAN sCT generation model used in this study achieves comparable results to the research models of Maspero et al.^18^ and Eckl et al.^15^

All methods showed global gamma index results above the clinically acceptable threshold, including outlier results, but method 2 was significantly lower than other methods. Gamma index results at the 2%/2‐mm threshold reported by Barateau et al.^20^ using a method similar to method 1 had a 1.1% lower global pass rate than presented in this study. Maspero^18^ and Eckl^15^ reported 4.0% and 5.0% lower global gamma pass rates, respectively, using a 2%/2‐mm tolerance, compared to method 4.

Methods 1 and 4 were the most dosimetrically accurate making them the most viable for clinical implementation. Methods 2 and 3 calculated significantly higher doses (up to 0.61%) than the rCT, especially in high dose regions. Although small, this could lead to regions of increased dose in recalculated treatment plans, unnecessarily triggering the ART pathway for a patient.

Dosimetric inaccuracies for methods 2 and 3 were also observed in several patients at the boundaries between high‐ and low‐mass density regions, resulting in a higher dose calculated in the trachea region than expected (Figure 3, third column). This is further evidence that the correction processes of methods 2 and 3 are imperfect. For example, method 2 has a sharp falloff of HU at the tissue/air boundary, whereas method 3 does not correctly assign density for air, giving it a much higher mass density than expected. These consistent errors in the assignment of air regions in methods 2 and 3 require further investigation to establish the clinical effect in the lung region.

Method 4 produced sCTs in the shortest time with the lowest variability as there was minimal subjective validation of the output required in this method, unlike the other methods where a review of the DIR or bulk densities created was necessary. The validation of this method should be performed upon clinical commissioning, and once confidence in the approach has been confirmed, it would not be necessary to perform further routine quality assurance as the model will not change. However, if patients are significantly different from the training cohort, this model would not be appropriate and results should be used with caution; it is therefore important to ensure that the training cohort is representative of the patients receiving treatment. The generation time did not include the time taken to train the model used in method 4 (∼100 h to run through 200 epochs). However, the training only needs to be performed once to generate the model and therefore does not affect the clinical pathway but should be considered when creating new models. As method 3 required a registration between the pCT and dCBCT, the time taken to generate an sCT using method 1 was included in method 3. It may also be possible to use a less detailed registration for method 3, thereby reducing the generation time. Improved processing hardware should also reduce the generation time of method 3, as well as method 4. Method 2 took the longest time to generate an sCT with the highest variance; the density threshold selection tool made fine adjustment of the thresholds difficult without affecting the other density thresholds. Optimizing the mass density threshold in one slice often resulted in a nonoptimal global mass density distribution, requiring a compromise to be made and therefore adding to the complexity and subjectivity of this method. Due to the subjectivity involved with the assignment of mass densities to CBCT intensity values, method 2 would not be suitable for automation unlike the other methods, and therefore the performance of this method in clinical situations would depend upon the experience and training of the operator. There is also a larger inherent inter‐ and intra‐operator error element in this method compared to others, although it is expected that more experience would result in quicker sCT generation times. Greater clinical experience in performing methods 1 and 3 may also reduce the time taken to generate their respective sCTs. Generation times were found to fluctuate with system utilization by up to 20 s for processing‐intensive methods (methods 3 and 4), and so the times reported here may not represent the generation times under clinical conditions.

These methods have only been assessed using a VMAT technique so should be validated for other treatment delivery techniques such as step and shoot intensity‐modulated radiation therapy that delivers a large amount of radiation through specific beam angles rather than across an arc, thereby making the accuracy of the electron density information more important. However, the authors expect that the change in a treatment technique will not make a significant impact on the overall dosimetric accuracy of the plan due to the low MAE in the HU that was measured across all methods.

A limitation of this study is that the dose is only calculated within the field of view of the CBCT; therefore, it is not representing the entire patient volume as would be required in a clinical pathway. Functionality within the TPS exists to create an examination that stitches the pCT outside of the CBCT external to the CBCT, which may partially address this issue; however, this would not include up to date anatomy. The limited field of view of the CBCT should be explored to determine the clinical dosimetric impact, with one solution being to acquire a larger field of view on the CBCT.

A further limitation of this study is that the ground truth comparison relies on a perfect DIR between the dCBCT and rCT; this is unlikely to be the case despite efforts to validate the registration accuracy qualitatively.^30^ Further investigation should be performed to understand the effect that imperfect DIRs had on this study, for example, performing a DIR between the pCT and rCT and comparing the results to method 1 in this study. The reliance on DIR in methods 1 and 3 needs to be investigated further for robustness with larger changes in the patient anatomy.

Further work should also be carried out to assess how these methods perform with greater differences in patient anatomy and patients with bolus, feeding tubes, and metal artifacts. These methods should also be evaluated on different anatomical sites where performance may change significantly.

As methods 3 and 4 are not yet available within the clinical version of RayStation, they cannot currently be implemented within the clinical pathway. Although the machine learning model was found to be the preferred method of sCT generation, there are still many challenges associated with its implementation, including ensuring that the sCTs produced are robust to all patients, mitigated by selecting a representative training cohort, and storing all the relevant data to the model such as the training and validation cohort and the parameters used to produce the model so as to be available for auditing. Discussions with clinicians, radiographers, and dosimetrists would also be required to determine the optimal way to incorporate this within the adaptive workflow. Ideally, this would be integrated into an automated pathway to perform a recalculation on the sCT generated from every CBCT captured, if significant changes are flagged, triggering intervention in the patient's treatment. However, this would require further work to validate the data transfer between the linac and the TPS as well as training for staff to use the new workflow and to be aware of any potential issues that may arise.

Due to the nature of this work assessing sCT generation within a commercially available TPS, as the software is updated, the performance of these methods may improve. Consequently, the results presented in this study should be continually reviewed, particularly for methods 3 and 4, which could be further optimized for imaging systems and imaging protocols used within different centers.

## 5 CONCLUSION

In this study, four methods of sCT generation were assessed in a commercial TPS. All methods showed sufficient dosimetric accuracy for clinical use, similar to results reported in literature where comparisons were possible.

Method 2, a bulk density assignment technique, and method 3, an HU correction and artifact reduction technique, behaved similarly regarding dosimetric accuracy as both were modified versions of the CBCT, with method 3 being more accurate due to the corrections applied in this method. Method 1, a deformed pCT to the dCBCT, and method 4, a machine learning technique, had the highest image quality and had excellent dosimetric accuracy with method 4 also having the shortest generation time.

Method 4, the machine learning method, is therefore the most suitable method for clinical implementation within the ART pathway, provided the model is trained with enough representative patient data.

## AUTHOR CONTRIBUTIONS

Guarantor of integrity of the entire study: Christopher J. O'Hara. Study concepts and design: Christopher J. O'Hara, David Bird, Richard Speight, and Bashar Al‐Qaisieh. Literature research: Christopher J. O'Hara. Experimental studies/data analysis: Christopher J. O'Hara, David Bird, and Richard Speight. Statistical analysis: Christopher J. O'Hara. Manuscript preparation: Christopher J. O'Hara. Manuscript editing: Christopher J. O'Hara, David Bird, Richard Speight, and Bashar Al‐Qaisieh.

## CONFLICT OF INTEREST

Supported by RaySearch Laboratories AB with a research license for the RayStation Treatment Planning System and support from developers.
